# Supplementary material for: The Differences in the Prevalence of Cardiovascular Disease, Its Risk Factors, and Achievement of Therapeutic Goals among Urban and Rural Primary Care Patients in Poland: Results from the LIPIDOGRAM 2015 Study
Source: J Clin Med. 2021 Nov 30;10(23):5656. doi: 10.3390/jcm10235656 (PMC8658414; doi:10.3390/jcm10235656)
Supplement: Supplementary file 1 [file jcm-10-05656-s001.zip › jcm-1436512-supplementary.pdf]

Supplementary 1 - LIPIDOGRAM2015 Investigators

Al-Shaer B., Andrusewicz W., Andrzejczuk-Rosa M., Anusz-Gaszewska E., Bagińska A., Balawajder P., Bańka G., Barańska-Skubisz E., Barbara Przyczyna B., Bartkowiak S., Bartodziej J., Bartosiewicz M., Basałyga M., Batyra A., Bąk A., Bednarz M., Bejnar K., Bernacki W., Betiuk-Kwiatkowska M., Biegaj S., Bień M., Bilski W., Biłogan M., Biruta-Pawłowska G. Biskup A., Błaszczyk B., Błaszczyk H., Błońska-Jankowska T., Bogacka-Gancarczyk B., Bojanowska M., Bonda E., Borowik-Skwarek J., Borowska J., Bruckner J., Brzostek J., Brzuchacz M., Budzyńska M., Bulzacka-Fugiel I., Bulzak J., Bunikowski K., Cebulska A., Celka T., Cempel-Nowak E., Chechliński W., Chludzińska A., Chmiel D., Chmielewska M., Cichy M., Ciemięga A., Ciepluch A., Cieszyńska I., Czajka B., Czapla B., Czerner M., Czerwińska B., Czuryszkiewicz W., Daleka E., Dawid Z., Dąbrowska M., Dąbrowska R., Dąbrowski D., Dąbrowski M., Demczyszyn K., Dębowska-Serwińska A., Dmochowski J., Dobrzecka-Kiwior J., Dolanowska E., Dolanowski H., Dołek P., Domagała M., Domański H., Doszel A., Duda D., Dudkowska M., Dudziuk B., Dybciak P., Dymanowski M., Dziadzio-Bolek L., Eicke M., El-Hassan H., Eremus A., Fąferek-Muller M., Figura-Roguska E., Fijałkowska-Kaczmarek I., Flis M., Florczak T., Florczuk M., Foryszewska-Witan E., Frydrych W., Fugiel A., Futyma E., Gaca-Jaroszewicz A., Gajdamowicz I., Ganczarski K., Gatnar A., Gers M., Głowacki A., Głód K., Godula J., Gołąb J., Gołębiewski M., Goszczyńska E., Gościcka K., Górna-Hajduga A., Górny E., Grabowska T., Grabowski R., Graczyk-Duda A., Gromow A., Grudewicz A., Gruszecka J., Gruszka A., Gryboś J., Grzebyk J., Grzechowiak A., Grzesiak D., Grześkowiak T., Guźla A., Hachuła G., Hawel B., Hiltawska H., Honkowicz E., Ignatowicz J., Imielski K., Iwaniura A., Jagieła-Szymala A., Jalć-Sowała M., Janczylik A., Janisz E., Janiszek M., Jankiewicz-Ziobro K., Januszewska K., Jaremek A., Jaros-Urbaniak A., Jarosz J., Jarosz P., Jasiński W., Jezierska-Wasilewska M., Jędraszewski T., Jędrzejowska A., Józefowicz R., Juźwin K., Kacprzak E., Kaczmarek-Szewczyk J., Kaczmarzyk M., Kandziora R., Kaniewski C., Karolak-Brandt L., Kasperczyk S., Kasperek-Dyląg E., Kedziora I., Kępa A., Kiciński J., Kielak-Al-Hosam J.,

Kiełczawa Ł., Kilimowicz P., Kitliński K., Kiwka T., Klein U., Klichowicz L., Klimowicz A., Klonowski B., Kmolek B., Kobyłko-Klepacka E., Kocoń A., Kolenda A., Kollek E., Kopeć M., Koper-Kozikowska B., Koralewska J., Korczyńska M., Korzeniewski M. T., Kosk A., Kotarski K., Kowalczyk E., Kowalczyk M., Kowalik I., Kozak-Błażkiewicz B., Kozik M., Kozłowska D., Kozłowska E., Kozłowska M., Kozubski T., Kózka K., Kraśnik L., Krężel T., Krochmal B., Król B., Król G., Król J., Królikowska T., Kruszewska H., Krygier-Potrykus B., Krystek W., Krzysztoń J., Kubicki T., Kuczmierczyk-El-Hassan A., Kuczyńska-Witek W., Kujda D., Kurowski A., Kurzelewska-Solarz I., Kwaczyńska M., Kwaśniak M., Kwaśniak P., Kwietniewska T., Łebek-Ordon A., Lebiedowicz A., Lejkowska-Olszewska L., Lentas M., Lesiewicz-Ksycińska A., Limanowski M., Łoniewski S., Łopata J. A., Łubianka B., Łukasiuk I., Łużna M., Łysiak M., Łysik B., Machowski Z., Maciaczyk-Kubiak J., Mackiewicz-Zabochnicka G. Magner-Krężel Z., Majda S., Malinowski P., Mantyka J., Marchlik E., Martyna-Ordyniec G., Marzec J., Marzec M., Matejko-Wałkiewicz R., Mazur M., Michalczak M., Michalska-Żyłka A., Michniewicz M.,Mika-Staniszewska D., Mikiciuk E., Mikołajczak T., Milewski J., Miller E., Misiaszek B., Mizik-Łukowska M., Młyńczyk-Pokutycka E., Mocek M., Moczała M., Morawska-Hermanowicz M., Moryc P., Moskal A., Moskal S., Moździerz A., Moździerz P., Mrozińska M., Mrozowicz K., Mróz G. Munia T., Mura A., Muras-Skudlarska M., Murawska E. Z., Murawski Ł., Murawski R., Musielak R., Nadaj K., Nagarnowicz W., Napierała R., Niedźwiecka M., Niemirski A., Nikiel J., Nosal M., Nowacki W., Nowak J., Nyrka M., Obst A., Ochowicz J., Ogonowska E., Oleszczyk M., Ołdakowski A., Ołowniuk-Stefaniak I., Ordowska-Rejman J., Orliński M., Osińska B., Ostańska-Burian A., Paciorkowska A., Paczkowska U., Paluch L., Pałka L., Paszko-Wojtkowska J., Paszkowska A., Pawlak-Ganczarska E., Pawlik W., Pawłowska I., Paździora M., Permiakow G. Petlic-Marendziak A., Piasecka T., Piaścińska E., Piktel A., Pilarska-Igielska A., Piotrkowska A., Piwowar-Klag K., Planer M., Plewa J., Płatkiewicz P., Płonczyńska B., Podgórska A., Polewska M., Porębska B., Porwoł P., Potakowska I., Prokop A., Przybylski J., Przybyła M., Psiuk H., Ptak K., Puzoń G. Rabiza N., Rachwalik S., Raczyńska E., Raniszewska M., Romanek-Kozik A., Rosa A., Rosa K., Rozewicz A., Rudzka-Kałwak J., Rusak J., Rutkowska D., Rybacki M., Rybińska D., Rycyk-Sadowska A., Rynda L.,

Rynkiewicz B., Sadowska-Krawczyk B., Sadowska-Zarzycka M., Sarnecka B., Sawalach-Tomanik E., Sidor-Drozd B., Siemieniak-Dębska M., Sieroń A., Siewniak-Zalewska B., Sikora A., Sitarska-Pawlina B., Skorupski J., Skrzypińska-Mansfeld I., Skubisz J., Skwarek R., Słodyczka M., Smentek M., Smolińska K., Solarz B., Sosnowska W., Sroka B., Stachura H., Stangreciak D., Staniak M., Stańczyk Z., Stańszczak-Ozga D., Startek E., Stefańczyk M., Stelmach R., Sternadel-Rączka E., Sternik M., Stępień J., Stocka J., Stokowska-Wojda M., Studler-Karpińska M., Suchorukow W., Sufryd W., Supłacz B., Sygacz J., Szczepański Ł., Szkandera J., Szłapa-Zellner J., Szydlarska D., Śliwa T., Śliwka J., Śmiejkowski Ł., Targońska A., Tesarska E., Tobiasz M., Tomaka J., Tomalska-Bywalec K., Tomiak E., Topczewski S., Trawińska A., Trela-Mucha L., Trojanowski D., Trzaskowska M., Trzcińska-Larska B., Trznadel-Mozul A., Ulanicka-Liwoch K., Urbanowicz M., Uthke-Kluzek A., Waczyński J., Walczak J., Warsz L., Wasyńczuk M., Wąchała-Jędras U., Wąsowicz D., Wczysła J., Wenda F., Werner-Kubicka E., Weryszko E., Węgrzynowska B., Wiaksa M., Wiankowski M., Wicherek A., Wieczorek R., Wiencek R., Wienzek-Tatara G., Wierzbicka B., Wierzbicki M., Wilczyńska B., Wilmańska D., Winiarski P., Wiszniewska-Pabiszczak A., Witkowska M. B., Witzling J., Wlaź A., Wojtkowiak I., Woydyłło J., Woźniak K., Wójtowicz A., Wrona J., Wrońska M., Wujkowska H., Wyrąbek J., Wysokiński O., Zakrzewski R., Zaleska-Zatkalik J., Zaleski J., Zalewska- Dybciak M., Zalewska E., Zalewska-Uchimiak B., Zawadzka-Krajewska J., Zawadzki J., Zieliński A., Zubrycka E., Żybort I., Żymełka M.

Table S1–Treatment used by LIPIDOGRAM 2015 study participants

	all			women			men		
	urban	rural	p	urban	rural	p	urban	rural	p
**Total n (%)**	7028 (51.21%)	6696 (48.79%)		4495 (51.7%)	4195 (48.3%)	0.11	2533 (50.3%)	2501 (49.7%)	0.11
**Treatment n (%)**									
**Lipid lowering**									
• fibrate	242 (3.44%)	222 (3.32%)	0.68	111 (2.47%)	102 (2.43%)	0.91	131 (5.17%)	120 (4.80%)	0.54
• simvastatin	657 (9.35%)	565 (8.44%)	0.06	411 (9.14%)	339 (8.08%)	0.08	246 (9.71%)	226 (9.04%)	0.41
• atorvastatin	821 (11.68%)	779 (11.63%)	0.93	510 (11.35%)	448 (10.68%)	0.32	311 (12.28%)	331 (13.23%)	0.31
• rosuvastatin	628 (8.94%)	632 (9.44%)	0.31	384 (8.54%)	358 (8.53%)	0.99	244 (9.63%)	274 (10.96%)	0.12
• ezetimibe	72 (1.02%)	83 (1.24%)	0.23	43 (0.96%)	42 (1.00%)	0.83	29 (1.14%)	41 (1.64%)	0.13
• other	113 (1.61%)	151 (2.62%)	0.01	75 (1.67%)	99 (2.36%)	0.02	38 (1.50%)	52 (2.08%)	0.12
• combined (use of ≥2 drugs)	125 (1.78%)	137 (2.05%)	0.25	64 (1.42%)	54 (1.29%)	0.58	61 (2.41%)	83 (3.32%)	0.05
**Antidiabetic**									
• gliclazide	83 (1.18%)	108 (1.61%)	0.03	51 (1.13%)	59 (1.41%)	0.26	32 (1.26%)	49 (1.96%)	0.0498
• glimepiride	102 (1.45%)	75 (1.12%)	0.09	58 (1.29%)	36 (0.86%)	0.05	44 (1.74%)	39 (1.56%)	0.62
• glipizide	28 (0.40%)	40 (0.60%)	0.10	11 (0.24%)	22 (0.52%)	0.03	17 (0.67%)	18 (0.72%)	0.84
• metformin	635 (9.04%)	588 (8.78%)	0.60	365 (8.12%)	317 (7.56%)	0.33	270 (10.66%)	271 (10.84%)	0.84
• acarbose	39 (0.55%)	39 (0.58%)	0.83	17 (0.38%)	25 (0.60%)	0.14	22 (0.87%)	14 (0.56%)	0.19
• insulin	205 (2.92%)	193 (2.88%)	0.90	109 (2.42%)	102 (2.43%)	0.98	96 (3.79%)	91 (3.64%)	0.78
• other	55 (0.78%)	65 (0.97%)	0.24	24 (0.53%)	42 (1.00%)	0.01	31 (1.22%)	23 (0.92%)	0.29
• combined (use of ≥2 drugs WITHOUT insulin)	158 (2.25%)	167 (2.49%)	0.34	88 (1.96%)	89 (2.12%)	0.59	70 (2.76%)	78 (3.12%)	0.46
• combined (use of insulin AND ≥1 drug)	95 (1.35%)	96 (1.43%)	0.68	47 (1.05%)	49 (1.17%)	0.59	48 (1.89%)	47 (1.88%)	0.97
**Antihypertensive**									
• ARBs	760 (10.81%)	729 (10.89%)	0.89	478 (10.63%)	457 (10.89%)	0.70	282 (11.13%)	272 (10.88%)	0.77
• ACEI	1498 (21.31%)	1430 (21.36%)	0.95	896 (19.93%)	805 (19.19%)	0.38	602 (23.77%)	625 (24.99%)	0.31
• CCB	579 (8.24%)	575 (8.59%)	0.46	333 (7.41%)	335 (7.99%)	0.31	246 (9.71%)	240 (9.60%)	0.89
• diuretics	1310 (18.64%)	1349 (20.15%)	0.03	835 (18.58%)	935 (19.90%)	0.12	475 (18.75%)	514 (20.55%)	0.11
• B-blockers	1422 (20.23%)	1315 (19.64%)	0.38	889 (19.78%)	801 (19.09%)	0.42	533 (21.04%)	514 (20.55%)	0.67
• other	357 (5.08%)	369 (5.51%)	0.26	226 (5.03%)	227 (5.41%)	0.42	131 (5.17%)	142 (5.68%)	0.43

• use of 1 drug	1551 (22.07%)	1392 (20.79%)	0.07	983 (21.87%)	864 (20.60%)	0.15	568 (22.42%)	528 (21.11%)	0.26
• combined (use of ≥2 drugs with FDC)	363 (5.17%)	349 (5.21%)	0.90	217 (4.83%)	189 (4.51%)	0.47	146 (5.76%)	160 (6.40%)	0.35
• combined (use of ≥2 drugs without FDC)	1423 (20.25%)	1437 (21.46%)	0.08	874 (19.44%)	872 (20.79%)	0.12	549 (21.67%)	565 (22.59%)	0.43

$p<0.05$ was considered statistically significant. ASA - acetylsalicylic acid, NOAC - novel oral anticoagulants, FDC - fixed-dose combination, ARB - angiotensin receptor blockers, ACEI - angiotensin-converting enzyme inhibitors, CCB - calcium channel blockers.

Figure S1–Therapeutic goals achievement according to 2018 ESH/ESC and 2019 ESC/EAS guidelines in the LIPIDOGRAM 2015 study.

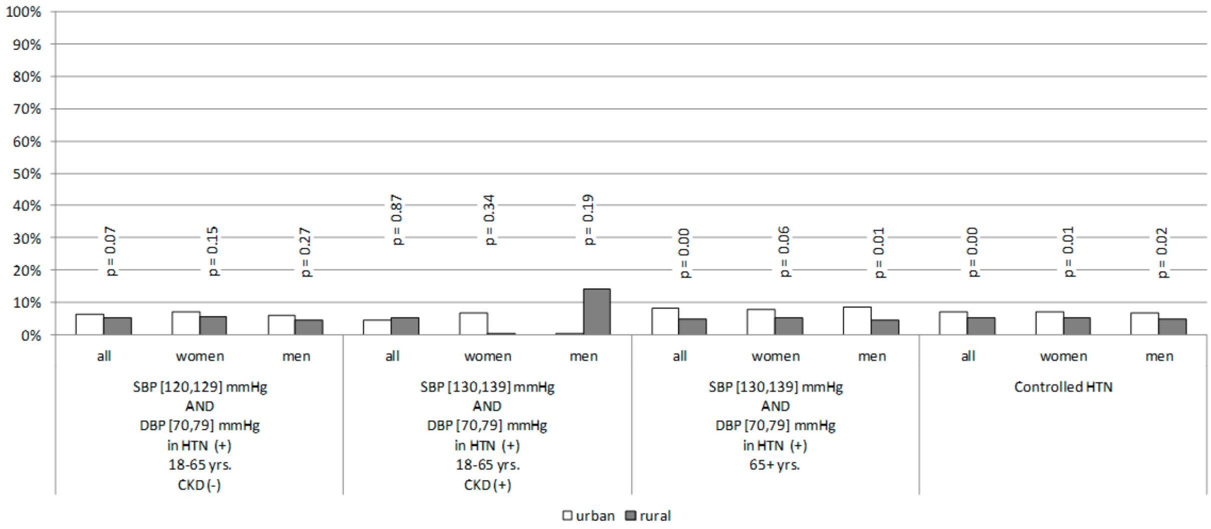

a. Percentage of hypertensive patients reaching treatment goals according to 2018 ESH/ESC guidelines [1].

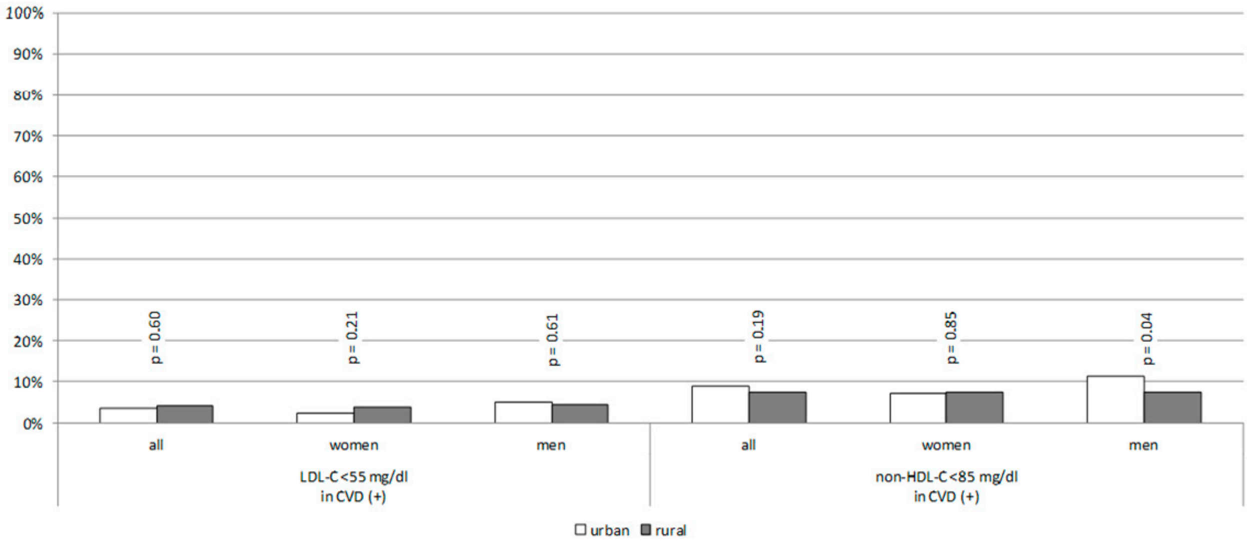

b Percentage of patients with cardiovascular diseases reaching treatment goals according to 2019 ESC/EAS guidelines [2].

Abbreviations:

HTN – hypertension, SBP – systolic blood pressure, DBP – diastolic blood pressure, CVD – cardiovascular disease, CKD – chronic kidney disease, CVD – cardiovascular disease, non-HDL-C – non high-density lipoprotein cholesterol, LDL-C – low-density lipoprotein cholesterol, TG – triglycerides

References

1. Williams B, Mancia G, Spiering W, et al. 2018 Practice Guidelines for the management of arterial hypertension of the European Society of Hypertension and the European Society of Cardiology: ESH/ESC Task Force for the Management of Arterial Hypertension. J Hypertens 2018;36:2284–309. doi:10.1097/HJH.0000000000001961
2. Mach F, Baigent C, Catapano AL, et al. 2019 ESC/EAS Guidelines for the management of dyslipidaemias: lipid modification to reduce cardiovascular risk. Eur Heart J 2020;41:111–88. doi:10.1093/eurheartj/ehz455
